# The impact of antidepressant use on MDMA fatalities: A matched case-control study using a post-mortem database

**DOI:** 10.1177/02698811261436573

**Published:** 2026-04-06

**Authors:** Kirsten L. Rock, Paul Rees, David Morgan, Caroline S. Copeland

**Affiliations:** 1 Centre for Pharmaceutical Medicine Research, Institute of Pharmaceutical Science, King’s College London, UK

**Keywords:** MDMA, antidepressant, fatality, case-control

## Abstract

**Background:**

3,4-methylenedioxymethamphetamine (MDMA) is being investigated as a new therapy for post-traumatic stress disorder (PTSD). With antidepressants as the first-line pharmacotherapy for PTSD, it is important to understand any drug-drug interactions between antidepressants and MDMA. This study aimed to investigate the association between antidepressant use and MDMA fatality using a drug-death database.

**Methods:**

A retrospective case-control study was performed using a dataset from the National Programme on Substance Use Mortality. Deaths reported between 1997 and May 2023 were extracted. Cases were defined as deaths with MDMA detected and were age- and sex-matched to controls, defined as deaths with no MDMA detected. Cases were analysed by post-mortem (PM) detection of antidepressants and prescription of antidepressants. Conditional logistic regression was performed, adjusted for confounders (polysubstance use and intentionality of death).

**Results:**

A total of 1328 cases and 5312 matched controls for the PM analysis and 840 cases and 3360 matched controls for the prescribed analysis were included. In the PM analysis, after adjusting for potential confounders, antidepressant use was less likely in MDMA fatalities compared to other drug-related deaths (adjusted OR [aOR] 0.595 [95% Confidence interval (CI) 0.491–0.722]). In the prescribed analysis, there was no significant association between the prescription of antidepressants and MDMA fatalities (aOR 0.838 [95% CI 0.688–1.021]).

**Conclusions:**

An inverse association was found between antidepressant use and MDMA fatality in this retrospective case-control study using a drug-death database.

## Introduction

3,4-methylenedioxymethamphetamine (MDMA) is under investigation as an alternative treatment for post-traumatic stress disorder (PTSD), principally for patients with poor response to current pharmacotherapy (Mitchell et al., 2023; Mithoefer et al., 2018). MDMA is a unique psychedelic drug due to its entactogenic effects that enhance feelings of empathy and closeness, increase sociability, and reduce fear responses (Feduccia and Mithoefer, 2018; Kamilar-Britt and Bedi, 2015). The aim of MDMA-assisted psychotherapy is to leverage the period of drug-induced neuroplasticity to change negative associations with traumatic memories and reduce symptoms of PTSD (Mitchell et al., 2023; Mithoefer et al., 2018). Some countries have updated regulations to allow for the prescribing of MDMA in specific cases, for example through Health Canada’s (2023) Special Access Program and the Authorised Prescriber pathway in Australia (TGA, 2023). In the United States, MDMA was granted a breakthrough therapy designation by the FDA in 2017 for its substantial improvement over available therapies for PTSD (Doblin R., 2017). However, in August 2024, the new drug application was ultimately rejected by the FDA, citing concerns about the methodology and conduct of the trials, stipulating that an additional Phase III trial is required for future consideration.

Psychiatric co-morbidity, and therefore concurrent use of psychotropic medications, is common in PTSD patients. Indeed, more than 50% of patients with PTSD have co-morbid depression (Rytwinski et al., 2013). Antidepressants are also recommended as first-line pharmacological treatment for PTSD. These include sertraline and paroxetine, two selective serotonin re-uptake inhibitors (SSRIs), and venlafaxine, a serotonin and noradrenaline re-uptake inhibitor (SNRI) (NICE, 2018). It is therefore important to consider any interaction between antidepressants, which are currently taken for PTSD treatment, and MDMA, a potential new therapy. The aim of this study was to investigate the association between antidepressant use and MDMA fatality using a drug-death database.

## Methodology

### Study design

A matched case-control study design was used to investigate the association between antidepressant use and MDMA fatality. Matching in case-control studies adjusts for confounders and improves study efficiency (Iwagami and Shinozaki, 2022). Here the confounding factors matched between the case and control groups were age and biological sex because of statistically significant differences in these variables in the unmatched dataset, with the distribution of cases in the younger age stratum compared to older controls (mean age: cases 30.0 ± 10.4, controls 40.5 ± 13.4, *p* < 0.001) and a larger proportion of males in the cases (cases 84% male, controls 73% male, *p* < 0.001). Cases were matched to controls in a 1:4 ratio. Four controls were chosen per case instead of matched pairs to improve the statistical power of the study (Grimes and Schulz, 2005). Exact matching was used for biological sex, and interval matching of ±3 years was used for age. Three years was selected as the interval because it was the smallest interval that allowed for all cases to be matched to four unique controls, given the sparsity of data in the younger age groups. By matching on these variables, cases and controls had similar distributions of these characteristics to increase the similarity of the exposure (antidepressant use).

The study was conducted according to Strengthening the Reporting of Observational Studies in Epidemiology guidelines (Supplemental Table 1) (von Elm et al., 2007).

### Data source

The National Programme on Substance Use Mortality (NPSUM, previously the National Programme on Substance Abuse Deaths) is a database of deaths related to psychoactive drug use in the UK. The NPSUM contains reports from England, Wales, and the surrounding islands since 1997, Northern Ireland from 2004, and Scotland from 2004–2011. Deaths are reported by coroners to the NPSUM if one or more psychoactive substances were detected at post-mortem (PM) by analytical toxicology testing and/or implicated in death, or if the decedent had a history of drug misuse or dependence. Around a third of all deaths are referred to a coroner for investigation, of which approximately 8% undergo a full post-mortem with toxicology (Ministry of Justice, 2025). The NPSUM data fields consist of decedent demographics, cause(s) of death and coroner’s conclusion, circumstances of death, toxicology reports detailing drugs detected, and medical history, including information relating to prescribed medicines in the 12 months preceding death.

The King’s College London Biomedical and Health Sciences, Dentistry, Medicine, and Natural and Mathematical Sciences Research Ethics Sub-Committee re-confirmed in August 2025 that NPSUM does not require ethics review, as all subjects are deceased.

#### Case identification

**Figure 1.**
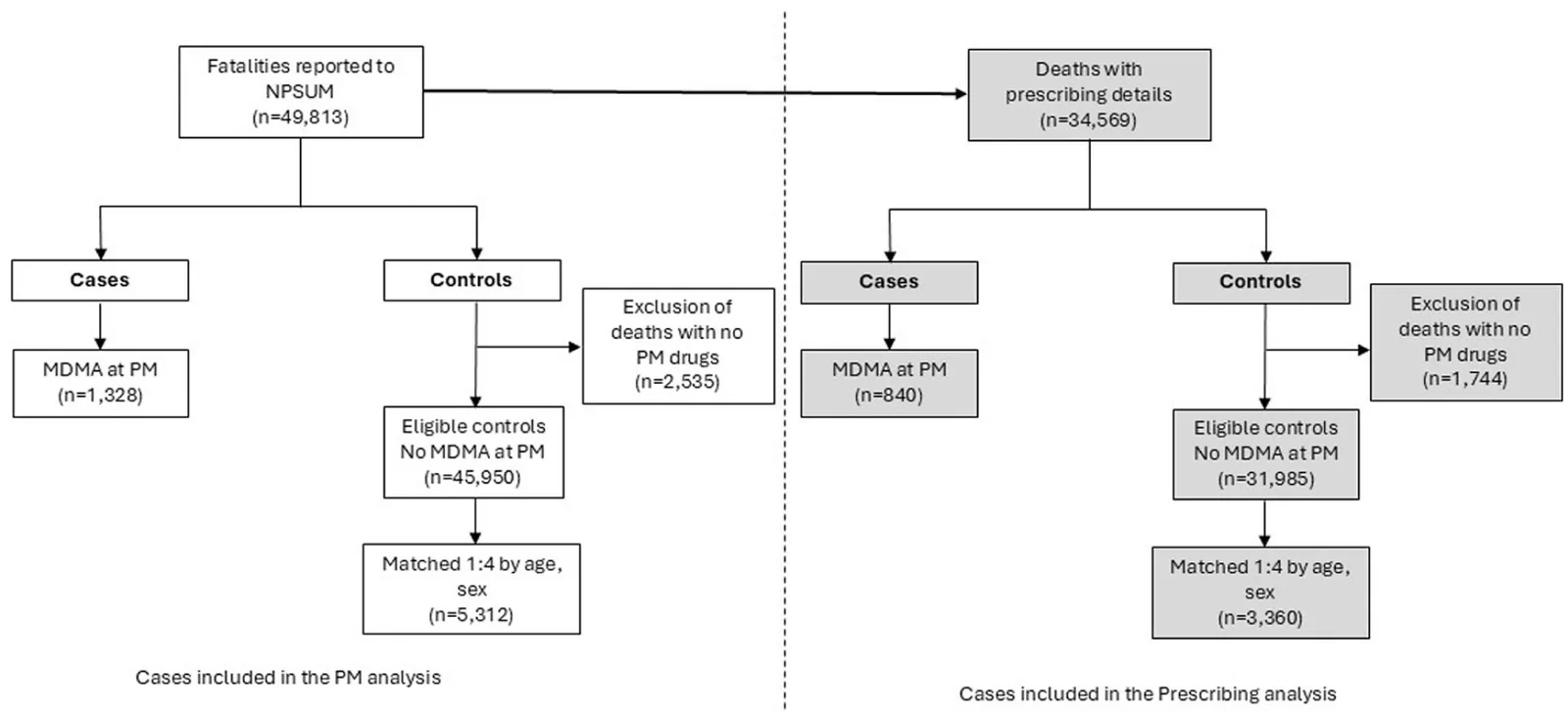
Flowchart of cases included in the study.

Any death received by the NPSUM from 1997 to the 1st of May 2023 was eligible for inclusion in the study. Cases were defined as having MDMA detected by PM toxicology testing and were extracted from the database using the numerical code assigned to the drug MDMA. Controls were defined as any death with no PM detection of MDMA. Controls were selected from the NPSUM database of 45,950 deaths and randomly matched to cases as described above (section ‘Study design’). The exclusion criteria were deaths with no toxicology testing performed or no drugs identified in human tissue. Figure 1 shows the flowchart of cases included in the final analysis.

In the UK, toxicology testing of PM samples of tissues (blood, urine, vitreous humour, gastric, liver, muscle) typically involves a drug screen using an immunoassay or a multi-analyte liquid chromatography mass spectrometry (LC–MS) screening technique (Rooney et al., 2023). Further confirmatory testing of drugs is performed using either an LC–MS/gas chromatography mass spectrometry (GC-MS) or high-resolution accurate mass analysis (HRAM), and alcohol analysis is performed using a dual column detector gas chromatography flame ionisation detector system (Rooney et al., 2023). The screening panel assesses samples for analytes of the antidepressant, opioid/opiate analgesic, antipsychotic, antihistamine, anxiolytics/hypnotic, non-opioid analgesic, anti-convulsant, antibiotic/fungal, and non-steroidal anti-inflammatory drug classes (Rooney et al., 2023). The panel also includes common recreational drugs such as cocaine, BZE, cocaethylene, amphetamine, methamphetamine-related drugs, piperazine derivatives, cannabinoids, ketamine, hallucinogens, benzodiazepine drugs, and a selection of new psychoactive substances prevalent on the illicit market at the time of testing (Rooney et al., 2023).

Antidepressants detected in the database were classified into their respective groups based on the NICE classifications (Table 1) (NICE, 2024, n.d.). Analysis of antidepressant use was performed first by antidepressant type and then by medication name. Only antidepressants with more than 30 detections or prescriptions are reported due to a lack of power to make meaningful statistical comparisons.

**Table 1.** Classification of antidepressants.

Antidepressant type	Medication name
TCA	amitriptyline, clomipramine, dosulepin, doxepin, imipramine, lofepramine, nortriptyline, trimipramine
MAOI	moclobemide, phenelzine, tranylcypromine
SNRI	duloxetine, venlafaxine
SSRI	citalopram, fluoxetine, fluvoxamine, paroxetine, sertraline, vortioxetine*
TeCA	mianserin, mirtazapine
Others	agomelatine, bupropion^, reboxetine, trazodone, tryptophan

TCA: Tricyclic antidepressant; MAOI: Monoamine oxidase inhibitor; SNRI: Serotonin and noradrenaline re-uptake inhibitor; SSRI: Selective serotonin re-uptake inhibitor; TeCA: Tetracyclic antidepressant.

* vortioxetine also 5-HT_3_ receptor antagonist.

^ off-label use in UK.

#### Potential confounding factors

The confounding variables considered in the multivariate regression model were other types of psychoactive drugs detected at PM/prescribed and the intentionality of death. Polysubstance use is a known risk factor for drug-related adverse events, including death (Pickens et al., 2022). PM detection of alcohol and cannabis, and PM detection or prescription of antipsychotics, hypnotic/sedatives, opioids, other stimulants, sedating antihistamines, and other psychoactive drugs (ones that did not fit into the previous categories) were coded as binary variables indicating their presence or absence in a case. The intentionality of death was also considered as a confounding variable because of MDMA deaths mainly concluded as unintentional (Schifano et al., 2003). Deaths were coded into the binary variables ‘unintentional’ or ‘intentional’ based on the coroner-reported conclusion on the death certificate.

#### Data cleaning

The database was cleaned prior to case matching. The PM drug fields were cleaned for accuracy of entries. Low alcohol levels (<10 mg/dL) were attributed to endogenous PM production rather than consumption of ethanol prior to death and were removed as a PM detection (O’Neal and Poklis, 1996). Antidepressants coded as ‘other antidepressant’ in the database were extracted and the original files manually searched and amended based on information provided in the file; for example, coding was changed to unspecified TCA for cases where ‘TCA antidepressant’ was noted in the original file or kept as ‘unspecified’ if no specific antidepressant was stated.

### Statistical analysis

The primary outcome measure was the incidence of antidepressant use in MDMA deaths, adjusted for potential confounders. Two analyses were conducted: first, the PM detection of antidepressants identified antidepressants that were taken around the time of death, evidenced by their presence in toxicological testing; second, the prescribing of antidepressants was considered to account for prescriptions dispensed in the 12 months before death that may not have been taken on the day of death (due to a variety of factors).

Descriptive statistics were calculated using frequencies (%) for categorical variables and median (interquartile range [IQR]) for continuous non-normally distributed variables. Unadjusted odds ratios (ORs) with a 95% confidence interval (CI) were calculated to examine bivariate associations between MDMA and antidepressants, either at PM or prescribed. Conditional logistic regression was performed to estimate the adjusted odds ratios (aORs) and 95% CI in the multivariate analysis with confounders added into the model. Conditional logistic regression is a statistical method that accounts for matching in the analysis by stratifying on matching factors (i.e. age and sex in this study) (Pearce, 2016).

A statistically significant association was defined as a 95% CI that did not cross 1. All statistical analyses were conducted using IBM© SPSS Statistics software (version 27; Armonk, NY, USA). GraphPad Prism (version 10.4.1; GraphPad Software, San Diego, CA, USA) was used to produce the Forest plots.

## Results

### MDMA-related death and antidepressants

In total, 1328 deaths with PM detections of MDMA were identified and extracted from 49,813 deaths reported to NPSUM (Figure 1). The case group consisted of 1114 men (83.9%) and 214 women (16.1%). When matching on age and sex in a 1:4 ratio, 5312 deaths in the control group were identified, which consisted of 4456 men and 856 women (same proportions as cases). The median age in both groups was 28 years-old (IQR 22–36).

The prescribing analysis excluded cases where it was unknown if the decedent was prescribed medications on the day of their death. There were 840 deaths with MDMA identified and extracted from 34,569 deaths where details of the prescribed medications were provided (Figure 1). The case group for the prescribing cohort consisted of 701 men (83.5%) and 139 women (16.5%). Three thousand three hundred and sixty deaths were identified for the control group, which consisted of 2804 men and 556 women (same proportions as cases). The median age in both groups was 28 years (IQR 22–37).

The frequencies of antidepressant use by type are presented in Table 2. The controls had larger proportions of antidepressant use as indicated by their PM detections and prescriptions than the cases. Note that in some deaths, more than one antidepressant was detected. For the confounding variables, cases had a larger proportion of other stimulants detected at PM and lower proportions of hypnotic/sedatives and opioids detected at PM and prescribed compared to controls (Table 2).

**Table 2.** Frequency of antidepressant types and confounding variables in cases and controls.

Variable	MDMA at PM *n* (%)	No MDMA at PM *n* (%)
PM detection		
Any antidepressant	190 (14.3)	1334 (25.1)
TCA	61 (4.6)	469 (8.8)
SSRI	78 (5.9)	568 (10.7)
SNRI	21 (1.6)	123 (2.3)
TeCA	52 (3.9)	357 (6.7)
Confounders		
Alcohol	543 (40.9)	2045 (38.5)
Antipsychotic	69 (5.2)	433 (8.2)
Cannabis	264 (19.9)	747 (14.1)
Hypnotic/Sedative*	384 (28.9)	2010 (37.8)
Opioid	485 (36.5)	4094 (77.1)
Other psychoactive drug	280 (21.1)	967 (18.2)
Other stimulant	725 (54.6)	1793 (33.8)
Sedating antihistamine	29 (2.9)	208 (3.9)
Intentionality^‡^	1187 (89.4)	4339 (81.7)
Prescribed		
Any antidepressant	185 (22.0)	1012 (30.1)
TCA	34 (4.0)	228 (6.8)
SSRI	90 (10.7)	498 (14.8)
SNRI	24 (2.9)	103 (3.1)
TeCA	39 (4.6)	254 (7.6)
Confounders		
Alcohol^^^	355 (42.3)	1311 (39.0)
Antipsychotic	71 (8.5)	443 (13.2)
Cannabis^^^	168 (20.0)	521 (15.5)
Hypnotic/Sedative*	98 (11.7)	775 (23.1)
Opioid	80 (9.5)	775 (23.1)
Other psychoactive drug	88 (10.5)	430 (12.8)
Other stimulant	8 (1.0)	19 (0.6)
Sedating antihistamine	5 (0.6)	65 (1.9)
Intentionality^‡^	746 (88.8)	2642 (78.6)

TCA: Tricyclic antidepressant; SNRI: Serotonin and noradrenaline re-uptake inhibitor; SSRI: Selective serotonin re-uptake inhibitor; TeCA: Tetracyclic antidepressant; PM: Post-mortem.

* BZD, barbiturate, Z-drug.

^ PM detection.

‡ Unintentional deaths stated.

### Association between MDMA and PM detection of antidepressants

In the bivariate PM analysis, deaths with MDMA detections were less likely to have a co-incident antidepressant detection, with an unadjusted OR of 0.498 (95% CI 0.422–0.588). The antidepressant types TCAs, SSRIs, and TeCAs also had statistically significant ORs (Table 3). The other antidepressant types had no significant association, though the number of detections was smaller, so comparisons had less power to detect any difference.

**Table 3.** ORs and aORs for PM detections of antidepressants in cases and controls.

PM detection	MDMA at PM (*n*)	No MDMA at PM (*n*)	Antidepressant vs. no antidepressant		Antidepressant vs. no antidepressant	
			OR	95% Cl	aOR	95% Cl
**Any antidepressant**	**190**	**1334**	**0.498**	**0.422–0.588**	**0.595**	**0.491–0.722**
**Any TCA**	**61**	**469**	**0.497**	**0.378–0.654**	**0.554**	**0.410–0.748**
Amitriptyline	45	324	0.540	0.393–0.742	0.628	0.443–0.890
Dosulepin	12	118	0.401	0.221–0.729	0.405	0.214–0.766
**Any SSRI**	**78**	**568**	**0.521**	**0.408–0.666**	**0.625**	**0.477–0.820**
Citalopram	29	249	0.454	0.308–0.670	0.575	0.378–0.876
Fluoxetine	18	132	0.539	0.328–0.886	0.655	0.383–1.120
Paroxetine	7	24	1.168	0.502–2.716	1.742	0.670–4.532
Sertraline	26	181	0.566	0.374–0.858	0.620	0.390–0.984
**Any SNRI**	**21**	**123**	**0.678**	**0.425–1.081**	**1.087**	**0.648–1.823**
Duloxetine	6	25	0.960	0.393–2.344	1.812	0.674–4.870
Venlafaxine	15	99	0.602	0.348–1.039	0.908	0.499–1.651
**Any TeCA** *	**52**	**357**	**0.566**	**0.420–0.761**	**0.742**	**0.532–1.035**
Mirtazapine	51	357	0.554	0.411–0.748	0.723	0.517–1.010
**Other**						
Trazodone	6	37	0.647	0.273–1.536	1.110	0.445–2.770

*Note.* Bold indicates antidepressant type. Blue-shaded box indicates statistical significance. TCA: Tricyclic antidepressant; SNRI: Serotonin and noradrenaline re-uptake inhibitor; SSRI: Selective serotonin re-uptake inhibitor; TeCA: Tetracyclic antidepressant; OR: Odds ratio; aOR: Adjusted odds ratio; Cl: Confidence interval; MMDA: 3,4-methylenedioxymethamphetamine; PM: Post-mortem.

* includes mianserin (*n* = 1).

**Figure 2.**
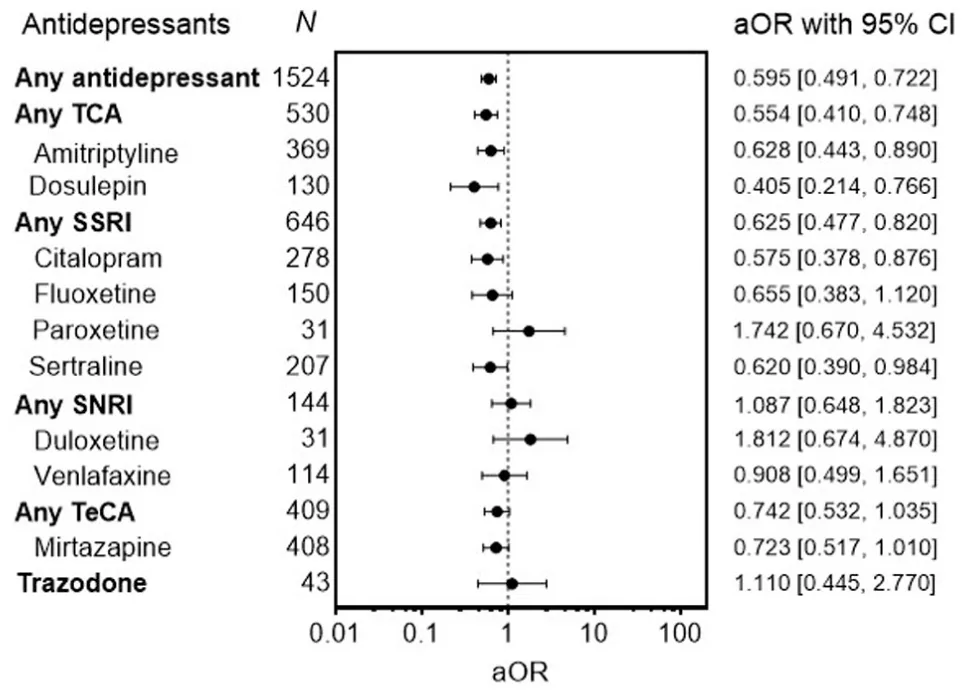
Adjusted odds ratios of antidepressants detected at post-mortem.

In the multivariate analysis controlling for confounders, the aOR for any antidepressant at PM remained significant at 0.595 (95% CI 0.491–0.722) (Figure 2 and Table 3). TCAs and SSRIs were associated with a lower co-incidence. For the TCAs, amitriptyline and dosulepin remained significant (amitriptyline aOR: 0.628 [95% CI 0.443–0.890]; dosulepin aOR: 0.405 [95% CI 0.214–0.766]). For the SSRIs, sertraline and citalopram remained significant (sertraline aOR: 0.620 [95% CI 0.390–0.984]; citalopram aOR: 0.575 [95% CI 0.378–0.876]).

Co-detection of antipsychotics and opioids was less likely in MDMA deaths, whereas cannabis and other stimulants were more likely to be detected in MDMA deaths (Supplemental Figure 1). MDMA deaths were 84% less likely to have opioids detected at PM (aOR: 0.156 [95% CI 0.134–0.181]) and nearly twice as likely to have other stimulants detected at PM (aOR: 1.831 [95% CI 1.590–2.107]). MDMA deaths were also more likely to be concluded as unintentional compared to other types of drug deaths.

### Association between MDMA and prescribed antidepressants

In the bivariate prescribed analysis, deaths with MDMA detections were less likely to have an antidepressant prescribed, with an unadjusted OR of 0.655 (95% CI 0.548–0.784). The antidepressant types TCAs, SSRIs, and TeCAs also had statistically significant ORs, and within each type only amitriptyline, citalopram, and mirtazapine were less likely to be prescribed in MDMA deaths (Table 4).

**Table 4.** ORs and aOR for prescribed antidepressants in cases and controls.

Prescribed	MDMA at PM (*n*)	No MDMA at PM (*n*)	Antidepressant vs. no antidepressant		Antidepressant vs. no antidepressant	
			OR	95% Cl	aOR	95% Cl
**Any antidepressant**	**185**	**1012**	**0.655**	**0.548–0.784**	**0.838**	**0.688–1.021**
**Any TCA**	**34**	**228**	**0.579**	**0.401–0.838**	**0.822**	**0.555–1.219**
Amitriptyline	21	151	0.545	0.343–0.866	0.771	0.474–1.256
Dosulepin	8	61	0.520	0.248–1.091	0.722	0.331–1.573
**Any SSRI**	**90**	**498**	**0.690**	**0.543–0.875**	**0.790**	**0.615–1.015**
Citalopram	30	187	0.628	0.424–0.931	0.735	0.492–1.098
Fluoxetine	30	140	0.852	0.570–1.273	0.946	0.624–1.435
Paroxetine	12	41	1.173	0.614–2.242	1.571	0.794–3.111
Sertraline	23	141	0.643	0.411–1.005	0.692	0.436–1.096
**Any SNRI**	**24**	**103**	**0.930**	**0.593–1.460**	**1.243**	**0.771–2.005**
Venlafaxine	19	81	0.937	0.565–1.553	1.230	0.722–2.095
**Any TeCA**	**39**	**254**	**0.595**	**0.421–0.841**	**0.726**	**0.504–1.045**
Mirtazapine	39	254	0.595	0.421–0.841	0.726	0.504–1.045
**Other**						
Trazodone	6	26	0.923	0.378–2.249	1.293	0.503–3.321

*Note.* Bold indicates antidepressant type. Blue-shaded box indicates statistical significance. Abbreviations: OR, Odds ratio; aOR, adjusted odds ratio; Cl, confidence interval; TCA: Tricyclic antidepressant; SNRI: Serotonin and noradrenaline re-uptake inhibitor; SSRI: Selective serotonin re-uptake inhibitor; TeCA: Tetracyclic antidepressant; MMDA: 3,4-methylenedioxymethamphetamine; PM: Post-mortem.

**Figure 3.**
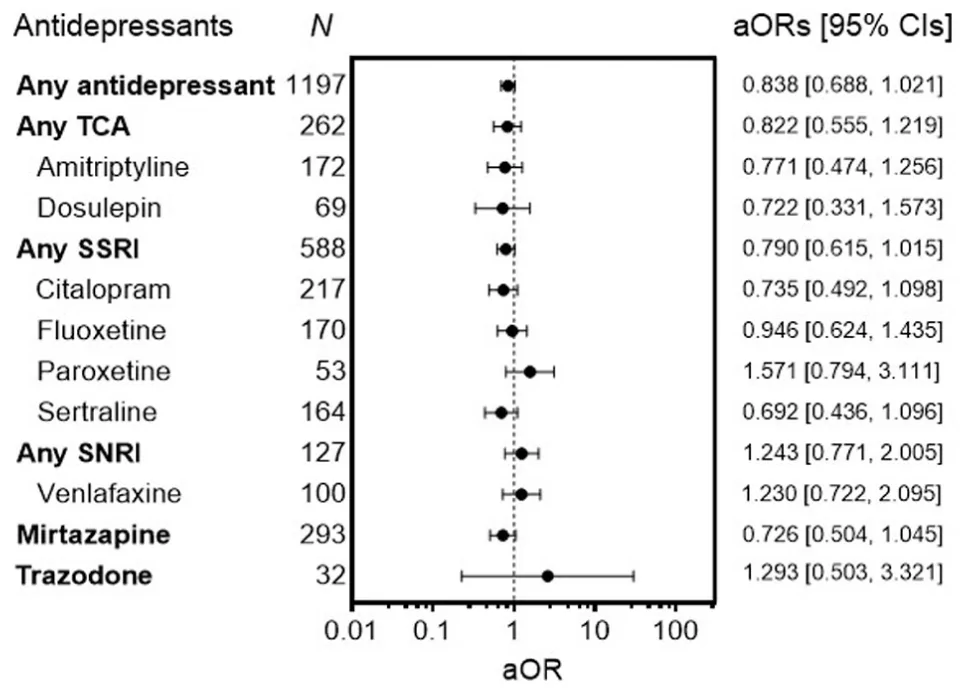
Adjusted odds ratios of prescribed antidepressants.

In the multivariate analysis controlling for confounders, none of the antidepressants remained significant (Figure 3 and Table 4).

The prescription of antipsychotics, hypnotic/sedatives, and opioids was less likely in MDMA deaths (Supplemental Figure 2). In contrast, cannabis and other stimulants were more likely to be co-detected or prescribed, respectively.

## Discussion

### Main Findings

In this retrospective case-control study, two analyses were performed to examine the association between both immediate concurrent antidepressant use (PM analysis) and long-term antidepressant use (prescribing analysis) with MDMA fatality. In the PM analysis investigating concurrent MDMA and antidepressant use, MDMA fatalities were 40% less likely to have co-detected antidepressants at PM compared to other drug-related deaths. In the prescribing analysis covering long-term antidepressant use, there was no significant association between prescribed antidepressants and MDMA fatalities.

Two contrasting behaviours of people who use MDMA recreationally have been previously reported in the literature. The first behaviour is people who intentionally take antidepressants in combination with MDMA to counteract the known adverse effects of MDMA. SSRI antidepressants have been used to blunt the ‘comedown’ or ‘hangover’ in the days after MDMA use, a phenomenon characterised by a depressed mood, anxiety, paranoia, and/or trouble sleeping caused by serotonin depletion (McCann and Ricaurte, 1993; Singh and Catalan, 2000). Antidepressants are also taken to attenuate the physiological effects associated with MDMA use, such as MDMA-mediated rises in body temperature, heart rate, and blood pressure (Farré et al., 2007; Liechti and Vollenweider, 2000; Price et al., 2022; Sarparast et al., 2022; Tancer and Johanson, 2007). Despite both being serotonergic drugs, antidepressant co-use may ameliorate MDMA-mediated adverse effects and toxicity, further evidenced in this study by a lower co-incidence of antidepressants in MDMA deaths. A previous study using data from the FDA Adverse Event Reporting System (FAERS) found a reduced risk of MDMA death with paroxetine and fluoxetine co-detection (Cohen et al., 2021). However, the same study also found an increased risk of MDMA death with citalopram and sertraline (Cohen et al., 2021), the two SSRIs that were less likely to be co-detected in MDMA deaths in this study. The second behaviour is individuals who are chronically prescribed antidepressants not taking their medication acutely during periods of MDMA use. Whilst there is evidence to suggest that antidepressants ameliorate the adverse effects of MDMA, it has similarly been reported that they can attenuate the intended euphoric effects of MDMA (Price et al., 2022; Sarparast et al., 2022). During times of recreational MDMA use, individuals may miss a dose of their antidepressant to avoid dampening or lessening their ‘high’ (Boeri et al., 2008; Edwards et al., 2022; Kruger et al., 2025; Whelan et al., 2024). Others may stop their prescription under the assumption that co-use may cause serotonin syndrome (Dobry et al., 2013). Alternatively, the dose of MDMA may be modified to offset their antidepressant prescription, either increasing the dose to overcome the ‘dampening’ effect, or reducing the dose to avoid perceived potential risks with co-use. This behaviour of individuals not taking their antidepressant prescription when using MDMA was investigated in the prescribing analysis of this study: where a decedent was listed as being prescribed an antidepressant (chronic antidepressant use) but had not taken it on the day of death (evidenced by a lack of post-mortem detection), there was a non-significant trend towards a lower co-incidence in MDMA deaths.

### Potential mechanism of reduced toxicity and clinical impact

MDMA is a substrate for the serotonin, noradrenaline, and dopamine transporters (SERT, NET, DAT, respectively) and binds to these transporters to be taken up into the axon terminal of the neuron (Capela et al., 2009; Green et al., 2003). Inside the neuron, MDMA disrupts the vesicular monoamine transporter protein to promote the release of stored serotonin neurotransmitters and reverses SERT function to facilitate the movement of intracellular serotonin out of the transporter (Capela et al., 2009). Both of these mechanisms increase extracellular serotonin concentration, thereby causing the subjective effects that are characteristic of MDMA use. The same physiology occurs at NET, causing the physical effects of MDMA and DAT, to which MDMA has a lower affinity (Green et al., 2003). As antidepressants act directly on monoamine transmitter systems or monoamine enzymes (Stahl, 2000), pre-treatment with antidepressants is hypothesised to block SERT, preventing uptake of MDMA into the axon terminal and subsequent release of serotonin.

In laboratory experiments, pre-treatment with an SSRI antidepressant (citalopram or fluoxetine) before MDMA administration offered protection against MDMA-induced damage to serotonergic neurons (Li et al., 2010; Piper et al., 2008; Sanchez et al., 2001). In two of the studies, the neuroprotective effect lasted until the final recorded timepoint, at 7 and 31 days, respectively (Li et al., 2010; Sanchez et al., 2001), but another showed a reduction in neuroprotection between the 1-week timepoint and the final timepoint at 10 weeks (Piper et al., 2008). Acute dosing of an SSRI appears to provide a neuroprotective effect when followed by MDMA, as described anecdotally by MDMA users and supported by laboratory experiments. The proposed mechanism of serotonergic blockade by SSRIs and, therefore, lower SERT availability for MDMA is further supported by the findings in this study because the two types of antidepressants that act as serotonin re-uptake inhibitors, SSRIs and TCAs (which have further activity at other receptors and neurotransmitters), were less likely to be co-detected in MDMA deaths.

A long-term antidepressant prescription may not offer improved protection over an acute dose, evidenced by no significant effect in the prescription sub-analysis of this study. Long-term antidepressant use results in the downregulation of serotonin receptors (Stahl, 2000). Although some protective effect may be conferred from a reduction in serotonin receptor density, this study suggests that the active inhibition of MDMA uptake by SSRI activity has a greater barrier to toxicity. Further research is needed, and future clinical trials can stratify participants by pre-treatment with antidepressants or previous antidepressant prescription to specifically look at the clinical impact of antidepressant co-use with MDMA-assisted therapy.

### Confounding variables

It is known that MDMA deaths are primarily unintentional (Schifano et al., 2003), and that polysubstance use is common among MDMA users (Soar et al., 2009), trends that were both reflected in the current dataset. Co-detection or prescription of stimulants was twice as likely to be detected in MDMA deaths. Concomitant use of MDMA with other stimulants with similar pharmacological actions, such as amphetamine and cocaine, potentiates the risk of adverse effects due to massively elevated serotonin levels. Serotonin toxicity, also known as serotonin syndrome, is a life-threatening adverse reaction that results from an excess of serotonin in the synapses (Makunts et al., 2022). It manifests physically as hyperthermia, delirium, muscle rigidity, and seizures, among other clinical symptoms that have been observed with multiple stimulant use (Makunts et al., 2022). Combined MDMA and other stimulant use presents a danger to MDMA users who should abstain from taking these drugs together to lower the risk of serotonin toxicity and possible death.

Although opioids had a smaller and therefore more significant aOR than antidepressants in the multivariate model, the populations of people who use antidepressants and those who use opioids drastically differ. There is a larger proportion of young adults aged 18–39 prescribed antidepressants compared to opioids, with an average of 13% and 6%, respectively, receiving prescriptions in England (Taylor et al., 2019). MDMA users also tend to be younger, with use prevalence highest for 16–34-year-olds (ONS, 2023), and indeed, the median age in this dataset was 28-years-old, indicating that people who use MDMA differ from those who use opioids. For these reasons, it is more probable that the small aOR of opioids is an artefact of low opioid prevalence in this population rather than a true protective effect against MDMA toxicity.

## Limitations

This study has some limitations. First, drug-related death reports are voluntarily sent to NPSUM. However, at the time of data extraction, approximately 92% of coronial jurisdictions were reporting to NPSUM. Therefore, whilst not every drug-related death is collated, the longitudinal nature of the database means that there was a large sample of cases and matched controls, which can improve statistical power for the conditional logistic regression models performed.

Although overall case and control group numbers were large, some of the less commonly detected or prescribed antidepressants had small sample sizes, resulting in wide CIs, and potentially failed to find a significant association. This was more pronounced within the prescription sub-analysis, where there were fewer cases (compared to PM detections) due to varying levels of information provided in the drug-death reports with respect to prescription medication history. In addition, antidepressants with insufficient sample sizes for adequate statistical power were excluded.

An analysis of PM blood concentrations of MDMA was attempted to compare deaths with only MDMA detected at PM to deaths with MDMA and antidepressants co-detected at PM. However, it was not possible to do a sufficiently powered comparison due to low numbers of cases where quantifications were performed with only MDMA and antidepressants as the contributory drugs to death.

Finally, case-control studies are unable to describe causal links in data, but despite this, case-control studies can be a useful tool for identifying risk factors and associations between the exposure and outcome variables that may not be readily apparent through visual inspection of datasets. Complementary laboratory experiments or further investigations into these trends can add to the evidence provided in the case-control study.

## Conclusion

Antidepressants were less likely to be detected in MDMA fatality in this retrospective case-control study using a drug-death database. The effect size was similar across all antidepressants, but strongest with SSRIs and TCAs; however, these types also had the largest sample sizes, therefore offering greater statistical power compared to types with fewer numbers. The implications of this study are two-fold: first, for patients with PTSD who take antidepressants and undergo MDMA-assisted therapy, there is a need for greater understanding of the impact of prescribed medication on the therapeutic and pharmacological effects of MDMA, as co-administration will unavoidably become more common following market approval of MDMA and its use in clinical practice. Second, in non-clinical settings, this study provides further evidence of the potential protective effect of antidepressants against MDMA toxicity for people who take antidepressants and use MDMA recreationally. These findings can be used to provide guidance on the neuroprotective potential of antidepressants and inform harm reduction strategies for MDMA users.

## Supplemental Material

sj-docx-1-jop-10.1177_02698811261436573 – Supplemental material for The impact of antidepressant use on MDMA fatalities: A matched case-control study using a post-mortem database Supplemental material, sj-docx-1-jop-10.1177_02698811261436573 for The impact of antidepressant use on MDMA fatalities: A matched case-control study using a post-mortem database by Kirsten L. Rock, Paul Rees, David Morgan and Caroline S. Copeland in Journal of Psychopharmacology
